# Hydrophobic Compounds Reshape Membrane Domains

**DOI:** 10.1371/journal.pcbi.1003873

**Published:** 2014-10-09

**Authors:** Jonathan Barnoud, Giulia Rossi, Siewert J. Marrink, Luca Monticelli

**Affiliations:** 1 IBCP, CNRS UMR 5086, Lyon, France; 2 Université Claude Bernard Lyon I, Lyon, France; 3 Dept of Physics, University of Genoa, Genoa, Italy; 4 Groningen Biomolecular Sciences and Biotechnology Institute and Zernike Institute for Advanced Materials, University of Groningen, Groningen, The Netherlands; Max Planck Institute for Biophysical Chemistry, Germany

## Abstract

Cell membranes have a complex lateral organization featuring domains with distinct composition, also known as rafts, which play an essential role in cellular processes such as signal transduction and protein trafficking. *In vivo*, perturbations of membrane domains (e.g., by drugs or lipophilic compounds) have major effects on the activity of raft-associated proteins and on signaling pathways, but they are difficult to characterize because of the small size of the domains, typically below optical resolution. Model membranes, instead, can show macroscopic phase separation between liquid-ordered and liquid-disordered domains, and they are often used to investigate the driving forces of membrane lateral organization. Studies in model membranes have shown that some lipophilic compounds perturb membrane domains, but it is not clear which chemical and physical properties determine domain perturbation. The mechanisms of domain stabilization and destabilization are also unknown. Here we describe the effect of six simple hydrophobic compounds on the lateral organization of phase-separated model membranes consisting of saturated and unsaturated phospholipids and cholesterol. Using molecular simulations, we identify two groups of molecules with distinct behavior: aliphatic compounds promote lipid mixing by distributing at the interface between liquid-ordered and liquid-disordered domains; aromatic compounds, instead, stabilize phase separation by partitioning into liquid-disordered domains and excluding cholesterol from the disordered domains. We predict that relatively small concentrations of hydrophobic species can have a broad impact on domain stability in model systems, which suggests possible mechanisms of action for hydrophobic compounds *in vivo*.

## Author Summary

Cell membranes consist of a variety of lipids and proteins with inhomogeneous lateral distribution, forming domains with distinct composition and properties. These domains play a fundamental role in a number of biological processes, and perturbing them can have important effects on cellular functions. Some chemicals with high affinity for lipid membranes perturb membrane domains, but the link between properties of the chemicals and domain perturbation is not understood. The mechanisms of domain perturbation are also not understood. In the present work we use molecular simulations of model membranes to understand the driving forces and the mechanisms of domain perturbation by different chemicals. We explore the effect of six hydrophobic compounds, all of them rather simple and common but with different size, shape, and properties. We find that all hydrophobic compounds alter the stability of domains, but not all of them in the same way. We identify two groups of compounds with opposite effects: aromatic compounds stabilize domains, while aliphatic compounds destabilize them. Simulations also allow us to visualize, for the first time, the mechanism of domain perturbation – which is very difficult to assess experimentally. Our findings on model membranes suggest possible mechanisms of action for hydrophobic chemicals in living cells.

## Introduction

Biological membranes are both chemically and structurally heterogeneous. The constituent lipids can self-organize in domains [1], which differ in chemical composition and in physical properties, including structural, dynamic, and elastic properties. Domains have a functional role in cells: membrane proteins partition preferentially to one specific domain (or to domain boundaries) and carry out their function correctly only when in the appropriate environment – as expressed by the raft concept [1]. Membrane lateral organization is involved in biological processes such as membrane fusion [2], [3], signal transduction [4], protein trafficking [5], and viral infection [6], [7]. Alterations of the membrane lateral organization have been identified in pathologies like allergies and the Alzheimer disease [8], and have been linked to the mechanism of action of general anesthetics [9], [10]. Understanding the determinants of domain stability *in vivo* is therefore of paramount importance in biomedical sciences. Yet, characterization of raft domains *in vivo* is challenging because of the small size of the domains, which are typically smaller than optical resolution [11]. In model systems (i.e., vesicles), instead, domains are usually larger and can even coalesce to yield macroscopic phase separation. For this reason, model systems are often used to study membrane lateral organization [11]. Among model systems, the most frequently used are ternary mixtures of cholesterol and two lipids with different melting temperatures, as they show liquid-ordered (L_o_) – liquid-disordered (L_d_) phase coexistence, similar to cell membranes [12].

Compounds with sufficiently high affinity for membranes can modulate biological function by virtue of membrane-mediated effects [13]–[15], including the alteration of membrane lateral organization. Recent studies have shown that, in model membranes, phase coexistence is affected by a variety of compounds. For instance, some lipids [16], vitamin E [17], and n-alcohols [10] destabilize phase separation in ternary lipid mixtures. On the contrary, transmembrane helical peptides [18], benzyl alcohol [17], and polystyrene [19] stabilize phase separation. It is unclear which chemical or physical properties of the solutes determine stabilization or destabilization of phase separation. Systematic studies on the effect of solutes on membrane lateral organization are lacking. Moreover, the mechanisms of stabilization and destabilization of domains are not understood.

In the present report, we describe the effect of different hydrophobic compounds on lipid mixing in phase-separated membranes. Hydrophobic compounds partition largely to the interior of lipid membranes, hence they do affect many membrane properties. Hydrophobic compounds are extremely common in commercial products and in the environment; for instance, they are used as fuels in combustion engines, as solvents in industrial processes, and as scaffolds in drugs. Also, they are building blocks for many industrial polymers and they are found in the atmosphere as pollutants (e.g., in products of incomplete combustion of fossil fuels). We determine the effect of hydrophobic compounds on phase separation using coarse-grained (CG) molecular dynamics (MD) simulations of L_o_-L_d_ phase-separated lipid membranes. We focus on six different hydrophobic solutes covering a wide range of sizes and a variety of chemical structures: cyclohexane, octane, hexadecane, benzene, C_60_ fullerene and polystyrene. All solutes partition to the interior of the membrane but, remarkably, they show very different lateral distributions. We identify two distinct groups with different lateral distributions, and we show that they have opposite effects on lipid mixing. Finally, we determine the mechanism of action for both groups of molecules.

## Results

### Lipid mixing: Effect of aromatic vs. aliphatic solutes

**Figure 1:**
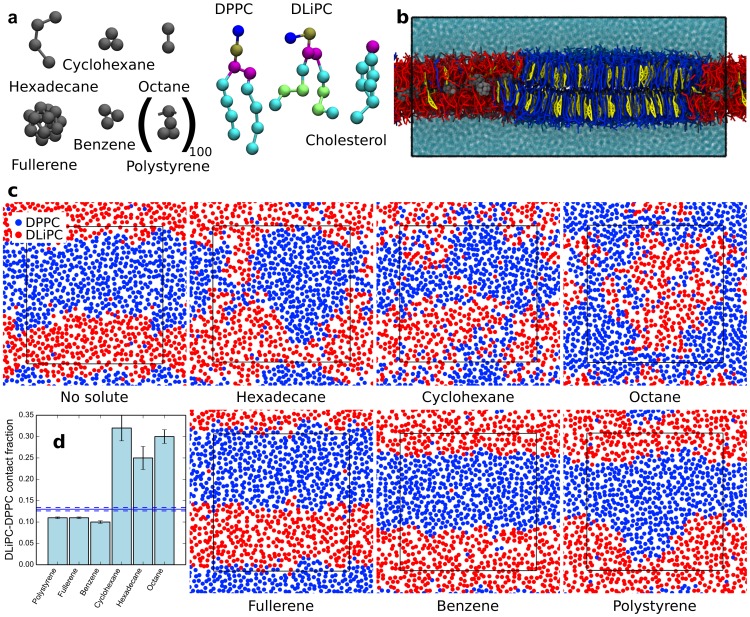
Effect of hydrophobic compounds on the stability of membrane domains. (a) MARTINI models of the hydrophobic solutes chosen for this study (colored in gray), phospholipids and cholesterol. (b) Side view of the phase separated membrane in the presence of fullerene at 295 K; DPPC is colored in blue, cholesterol in yellow, and DLiPC in red. (c) Top view of membrane systems with and without solutes (295 K, high solute concentration). Only one leaflet is displayed, and only one particle per lipid is shown (phosphate group), in red for DLiPC and in blue for DPPC. Solutes, cholesterol, and water are omitted for clarity. (d) DLiPC-DPPC contact fraction for the same systems; the solid blue line is for the case without solutes, the dashed lines are error estimates.

We used the MARTINI coarse-grained (CG) force field [20], [21] to simulate model membranes consisting of dipalmitoyl-phosphatidylcholine (DPPC), dilinoleyl-phosphatidylcholine (DLiPC), and cholesterol, at 42∶28∶30 molar ratio. At a temperature of 295 K, in the absence of solutes, the membrane showed phase separation into a liquid-ordered (L_o_) domain, comprising mostly DPPC and cholesterol, and a liquid-disordered (L_d_) domain, comprising mostly DLiPC, as reported previously [22]. Due to periodic boundary conditions used in the simulations, the domains organized in stripes along one axis of the box (Fig. 1). These stripes persisted during the simulation, yet the interfaces were dynamic and lipid molecules exchanged between the L_o_ and L_d_ phase.

We then carried out simulations of the same membrane in the presence of six different hydrophobic solutes: octane, hexadecane, cyclohexane, benzene, fullerene, and polystyrene. These solutes are chemically diverse, as they include linear alkanes of different size, a cyclic alkane, a common aromatic hydrocarbon, a well-known carbon nanoparticle, and a common industrial polymer. For each solute, we performed two simulations at low solute concentration (3.3% solute/lipid molar ratio) and two simulations at high solute concentration; due to the very different molecular weight of the solutes, for the high concentration we chose to use a common solute/lipid mass ratio of 4.8%. For polystyrene, we only considered simulations at high concentration. Additional simulations where performed for some solutes (see Table 1 for the complete list of simulations). Partition of the six solutes to the interior of lipid membranes is thermodynamically highly favorable, as shown in several previous studies [19], [23]–[25], but the time scales for permeation depend largely on the size of the particles: small molecules penetrate within a few nanoseconds [24] while large polymer particles require microseconds or more [19]. Since our interest was not in the kinetics of permeation but in the effect of the solutes on the membrane, we decided to start all simulations with the solutes placed inside the membrane, homogeneously distributed in the membrane plane.

**Table 1:** List of simulations performed.

Molecule	#mols	Temperature (K)	Mass ratio (%)	Molar ratio (%)	Duration (µs)*	DLiPC-DPPC contact fraction (*f*_mix_)	Solute-DLiPC contact fraction	Cholesterol-DLiPC contact fraction
None	0	295	0	0	30+10	0.13±0.004	-	0.11±0.015
None	0	305	0	0	10	0.20±0.017	-	0.13±0.003
None	0	315	0	0	10	0.25±0.029	-	0.16±0.010
None	0	325	0	0	10	0.34±0.017	-	0.20±0.004
Octane	64	295	0.56	3.29	24+20	0.15±0.004	0.51±0.006	0.11±0.003
Octane	276	295	2.43	14.20	7	0.24±0.005	0.44±0.003	0.14±0.002
Octane	547	295	4.81	28.14	25+15	0.30±0.016	0.41±0.002	0.15±0.010
Octane	743	295	6.53	38.22	6	0.33±0.013	0.39±0.001	0.16±0.003
Hexadecane	64	295	1.12	3.29	28+19	0.14±0.006	0.52±0.012	0.11±0.003
Hexadecane	276	295	4.81	14.20	15+21	0.25±0.027	0.43±0.013	0.13±0.006
Hexadecane	547	295	9.53	28.14	10	0.31±0.004	0.42±0.003	0.15±0.001
Hexadecane	743	295	12.95	38.22	10	0.30±0.009	0.44±0.013	0.14±0.005
Cyclohexane	64	295	0.41	3.29	15+10	0.14±0.010	0.52±0.011	0.10±0.002
Cyclohexane	276	295	1.79	14.20	6	0.25±0.003	0.44±0.020	0.13±0.015
Cyclohexane	547	295	3.54	28.14	10	0.28±0.002	0.42±0.001	0.12±0.001
Cyclohexane	743	295	4.81	38.22	22+19	0.32±0.030	0.40±0.002	0.13±0.010
Cyclohexane	743	325	4.81	38.22	20+20	0.48±0.004	0.36±0.001	0.24±0.002
Benzene	64	295	0.38	3.29	20+21	0.12±0.003	0.93±0.004	0.09±0.004
Benzene	800	295	4.81	41.15	24+13	0.10±0.003	0.90±0.004	0.03±0.001
Benzene	800	325	4.81	41.15	20+13	0.16±0.003	0.82±0.003	0.06±0.001
Benzene**	800	325	4.81	41.15	7	0.17±0.011	0.79±0.007	0.08±0.003
Benzene***	800	325	4.81	41.15	4.5	0.18±0.021	0.80±0.019	0.07±0.009
Fullerene	64	295	3.55	3.29	23+20	0.11±0.003	0.97±0.001	0.08±0.002
Fullerene	87	295	4.83	4.48	23+18	0.11±0.002	0.96±0.007	0.07±0.002
Fullerene	87	325	4.83	4.48	25+21	0.20±0.004	0.95±0.002	0.11±0.007
Polystyrene	6	295	4.81	0.31	10+10	0.11±0.002	0.94±0.001	0.06±0.004
Polystyrene	6	325	4.81	0.31	10+10	0.15±0.001	0.94±0.001	0.07±0.001

* When 2 numbers are reported, they refer to 2 independent simulations of the same system.

** Modified force field, with stronger interactions between cholesterol and aromatics (SC4-SC1 interaction set to ε = 3.5 kJ/mol, instead of ε = 3.11 kJ/mol).

*** Simulation starting from a mixed membrane (starting configuration taken from the last frame of the simulation in the presence of cyclohexane at 325 K).

To quantify phase separation, we calculated the DLiPC-DPPC contact fraction, *f*_mix_, defined as the fraction of DLiPC-DPPC contacts over the total number of contacts of DLiPC with all phospholipids (therefore not including cholesterol; see Methods). The DLiPC-DPPC contact fraction will tend to 0 at complete phase separation and will reach 0.61 at ideal mixing (equaling the DPPC molar fraction with respect to phospholipids only). In the absence of solutes, *f*_mix_ was 0.13±0.004, indicating strong phase separation. Addition of a small concentration of hydrophobic solutes had a minor effect on the DLiPC-DPPC contact fraction (0.11<*f*_mix_<0.15, depending on solute type; see Table 1). Yet two trends were distinguishable: octane, hexadecane, and cyclohexane caused an increase in lipid mixing, while benzene, fullerene, and polystyrene caused a slight decrease in lipid mixing. These trends were more evident at high solute concentration: *f*_mix_ reached 0.25–0.32 with the first group of compounds, and decreased to 0.10–0.11 with the second group (see Table 1). Visual inspection of the trajectories showed significant mixing (although not ideal mixing) in the presence of octane, hexadecane or cyclohexane, while domains were clearly separated in the presence of benzene, fullerene, or polystyrene (Fig. 1).

**Figure 2:**
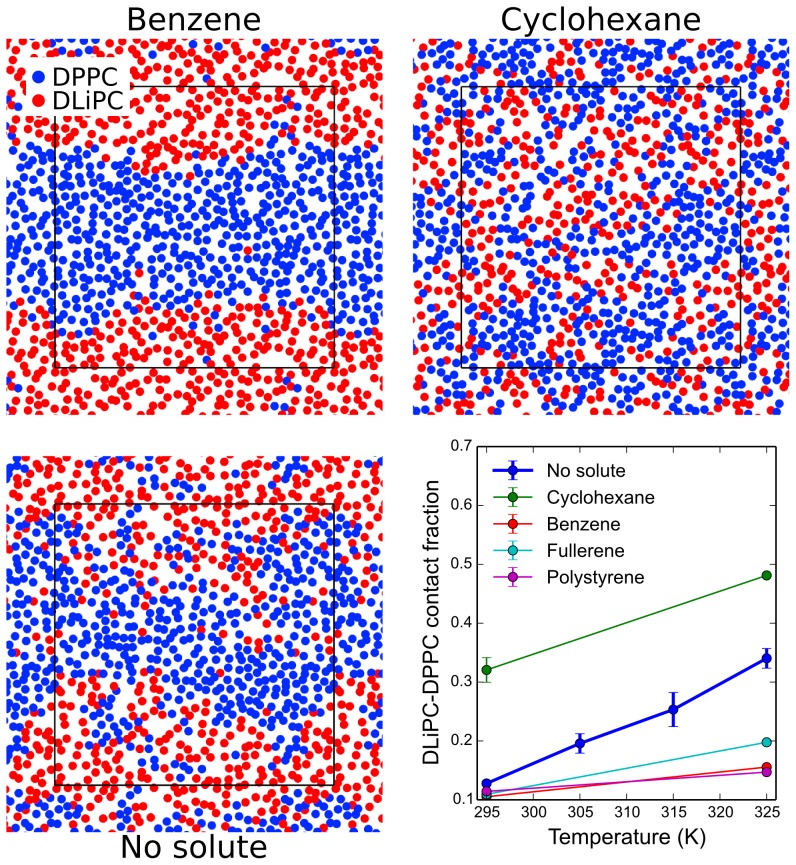
Thermal stabilization of phase separation. Snapshots of a single leaflet from simulations of systems with high concentration of benzene or cyclohexane, and with no solute, at 325 K. Colors are the same as in Fig. 1. Bottom right: DLiPC-DPPC contact fraction as a function of temperature from simulations without solutes and with high concentration of solutes. Thermal stabilization of phase separation is evident for fullerene, benzene, and polystyrene.

The demixing effect induced by benzene, fullerene, and polystyrene appeared weaker than the striking mixing effect of octane, hexadecane, and cyclohexane. This is because the reference membrane was already phase-separated at 295 K. To assess domain stabilization by benzene, fullerene, and polystyrene, we carried out simulations at higher temperature. At the temperature of 325 K the system without any solute was no longer phase-separated, with *f*_mix_ = 0.34±0.02 (Fig. 2). Remarkably, the membrane remained clearly phase-separated in the presence of benzene, fullerene, and polystyrene, and the increase in lipid mixing at higher temperature was minor (Fig. 2). The DLiPC-DPPC contact fraction was only 0.20±0.004 in the presence of fullerene at 325 K, and even less with benzene and polystyrene (Table 1). In contrast, in the presence of cyclohexane, lipids were already rather mixed at 295 K (at high concentration) and they mixed more at 325 K.

In summary, we observe a strong effect of all hydrophobic molecules on the stability of domains in phase-separated membranes, and we identify two groups of compounds with opposite effects on domain stability. The only obvious chemical property common within each group appears to be aromaticity (or the lack of it): all aromatic compounds promote lipid demixing, while all aliphatic compounds promote lipid mixing. What are, then, the mechanisms leading to such different effects on phase separation?

### Aliphatic solutes favor lipid mixing by acting as linactants

**Figure 3:**
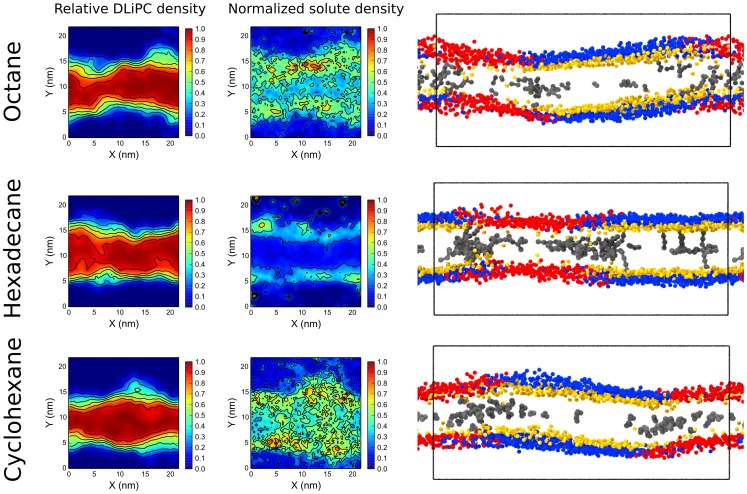
Aliphatic compounds act as linactants. Localization of DLiPC lipids and aliphatic solutes (averaged over the last 100 ns of simulation at low solute concentration, 295 K) expressed as relative density 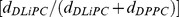 and normalized solute density 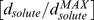. Right panels: snapshots from the same simulations, side view. Only one particle per lipid is shown; phosphate group is colored in red for DLiPC and in blue for DPPC, cholesterol hydroxyl group is in yellow, solutes in gray.

To understand the driving forces for solute-mediated alterations of membrane lateral organization, we analyzed the spatial distribution of each solute within the membrane by calculating the solute-DLiPC contact fraction (Fig. 3), defined as the number of contacts the solute makes with DLiPC over the number of contacts it makes with all phospholipids (see Methods). If the solute is ideally mixed, the solute-DLiPC contact fraction will be equal to the molar fraction of DLiPC with respect to all phospholipids, i.e., 0.39; lower values indicate that the solute makes contacts preferentially with DPPC, while higher values indicate that the solute makes contacts preferentially with DLiPC. As shown above, high concentrations of aliphatic solutes caused lipid mixing; the solute-DLiPC contact fraction in those systems was close to 0.39, as expected – lipid mixing is concurrent with solute mixing. More interesting is the behavior at low solute concentration (at 295 K), when the domains remain phase-separated. At low concentration, aliphatic solutes showed a preference for DLiPC. However, this preference was small, with solute-DLiPC contact fractions close to 0.5, indicating that the solute made the same number of contacts with DPPC and DLiPC. Such situation occurs if the solute is either found in both phases (with a mild preference for DLiPC), or if it lies at the interface between them. Analysis of the density landscapes of the different components indicated that aliphatic solutes distribute preferentially at the interface between the domains. The preference was very clear for hexadecane, and it was observable also for octane and cyclohexane (Fig. 3). Such preferential distribution suggests that aliphatic compounds act as linactants.

**Figure 4:**
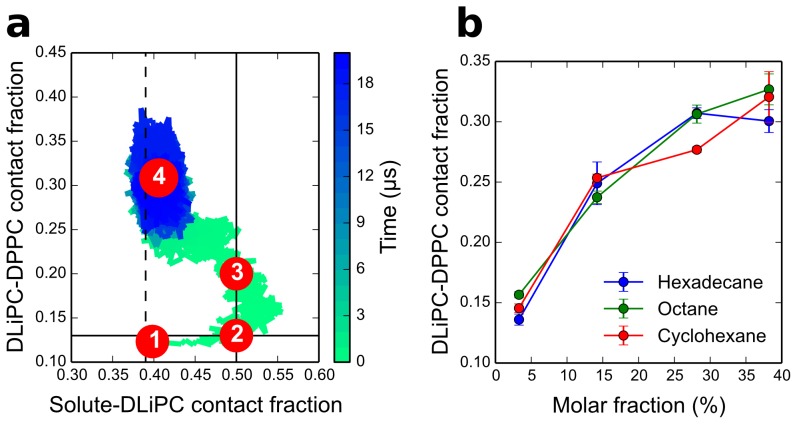
Mechanism of action and thermodynamics of linactants. (a) Mechanism of action of octane on phase-separated membranes: lipid mixing (expressed as DLiPC-DPPC contact fraction) vs. solute distribution (expressed as solute-DLiPC contact fraction) as a function of simulation time (represented with a color scale, from green to blue). The vertical solid line marks the solute-DLiPC contact fraction of 0.5 (solute mostly at the interface). The vertical dashed line marks a solute-DLiPC contact fraction of 0.39 (ideal mixing of the solute). The horizontal solid line indicates the DLiPC-DPPC contact fraction in the absence of solute. The numbers in red circles refer to specific times during the simulation: (1) t = 0 ns, (2) t = 40 ns, (3) t = 300 ns, (4) t = 20 µs. (b) Lipid mixing (expressed as DLiPC-DPPC contact fraction) as a function of linactant molar fraction at 295 K.

To understand the mechanism of action of aliphatic compounds in more detail, we plotted the DLiPC-DPPC contact fraction vs. solute-DLiPC contact fraction as a function of simulation time. Figure 4A shows the results for one simulation with octane at high concentration at 295 K. Initially, by design, octane was evenly distributed in the membrane, and the membrane was phase-separated (point 1 in the figure). During the first 40 ns, octane moved to the interface without affecting phase separation (1→2). Once octane molecules reached the interface, they remained there while the lipids started mixing (2→3). After about 300 ns, the domains started to become blurry (3). While phase separation disappeared, octane mixed as well (3→4). Finally, both the lipids and the solute were mostly mixed (4). A very similar behavior was observed also with hexadecane and cyclohexane, although the detailed kinetics was different (Fig. S1). The sequence of events indicates clearly that all aliphatic compounds act as linactants, first moving towards the L_d_-L_o_ interface and then destabilizing phase separation.

While the general mechanism of action was similar for all aliphatic compounds (Fig. S1), the kinetics of mixing depended on the nature and on the concentration of the solute: cyclohexane induced mixing faster than octane and hexadecane; also, in the presence of cyclohexane the transition (2→3) started before the solute-DLiPC contact fraction reached 0.5, i.e., before all the solute reached the interface. On the contrary, the time scale for mixing was longer in simulations with hexadecane; compared to octane and cyclohexane, hexadecane showed a higher affinity for unsaturated lipids.

Despite differences in the kinetics of lipid mixing, the extent of lipid mixing was remarkably similar for all aliphatic solutes at all concentrations, once concentrations were expressed as molar fractions (Fig. 4B). This indicates that the chemical potential of each lipid in the L_o_ and L_d_ phase did not depend on the type of aliphatic solute. In other words, the thermodynamics of lipid mixing was surprisingly independent of the nature of the solute.

### Aromatic compounds favor phase separation by redistributing cholesterol

**Figure 5:**
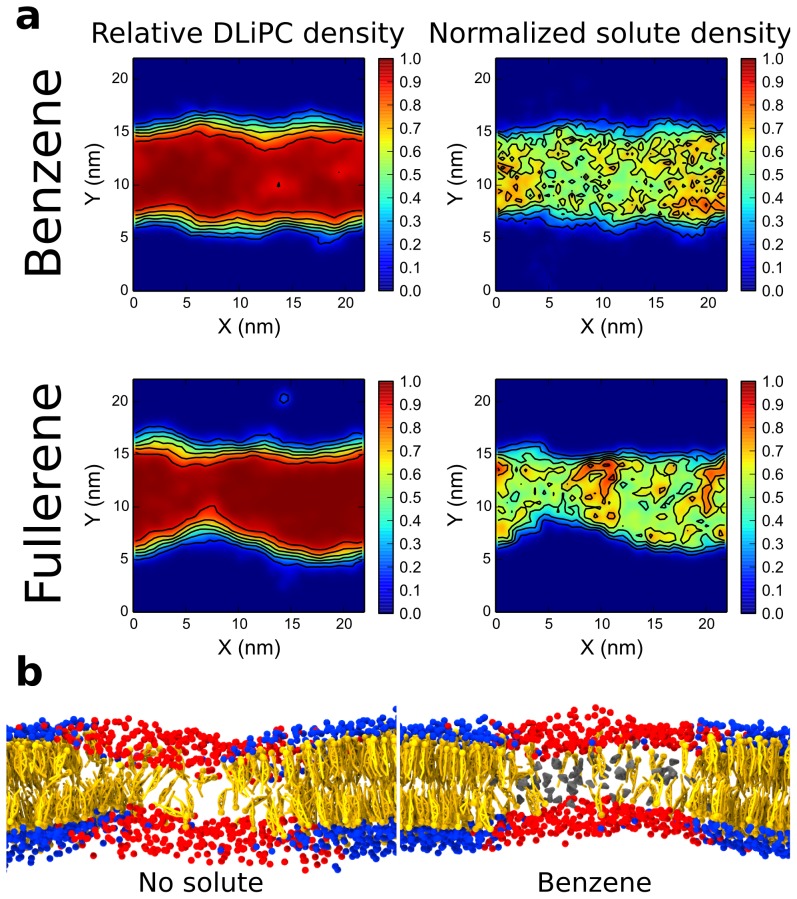
Mechanism of action of aromatic compounds. (a) Lipid and solute lateral distribution at 325 K, with high concentration of solute, expressed as relative DLiPC density normalized solute density. Aromatic solutes co-localize with unsaturated lipids. (b) Close-up view of the membrane centered on the L_d_ phase, in a system without solute (left) and in the presence of benzene (gray); cholesterol molecules are highlighted in orange. Benzene is found approximately at the same location as cholesterol.

In contrast to aliphatic compounds, aromatic solutes such as benzene, fullerene, and polystyrene, stabilized the L_o_-L_d_ phase separation. How did aromatics stabilize phase separation? To understand the underlying mechanism, we analyzed solute distribution in the membrane. Solute-DLiPC contact fractions for all aromatic compounds were close to 1, indicating a strong preference for the L_d_ phase, as also confirmed by density landscapes (Fig. 5). An obvious potential mechanism to promote phase separation involves changes in the properties of the L_d_ phase, where aromatics lie. For example, thinning of the L_d_ phase would lead to an increase in thickness mismatch between the L_d_ and L_o_ domains, favoring phase separation. However, we found that all aromatic solutes caused an increase in the thickness of the L_d_ domain (Fig. S3). As a result, the thickness mismatch between the two phases was actually reduced by these solutes, not increased. The largest reduction in thickness mismatch was observed in case of polystyrene, amounting to about 0.2 nm. Clearly changes in the thickness of the L_d_ phase cannot explain the effect of aromatic compounds.

An alternative hypothesis is that aromatic solutes compete with the (small) fraction of cholesterol that resides in the L_d_ phase. Visual inspection of the trajectories suggested that aromatic solutes replaced cholesterol in the L_d_ phase (Fig. 5). Cholesterol-DLiPC contact fraction showed that few cholesterol molecules partitioned to the L_d_ phase, both with and without added solutes. Yet, in the presence of aromatic compounds, the presence of cholesterol in the DLiPC-rich phase was significantly reduced, particularly at high temperature (see Table 1). We conclude that aromatic solutes, by partitioning into the L_d_ domain, provide an additional driving force for cholesterol to enter the L_o_ phase. As a result, the difference in order between the domains increases even further, and domain segregation becomes stronger.

Since the mechanism of action of aromatic compounds appeared to involve the displacement of cholesterol from the L_d_ phase, we verified that this result does not depend strongly on the particular choice of the cholesterol-aromatic interaction. We carried out additional simulations with a modified force field, in which the strength of cholesterol-aromatic interaction was increased (see Methods for the details). We found that phase separation was about the same as with the original force field (Fig. S4): in the presence of benzene, DLiPC-DPPC contact fraction was 0.17±0.01, very similar to the contact fraction obtained with the regular force field in the same conditions (0.16). Moreover, solute-DLiPC and cholesterol-DLiPC contact fractions calculated with the original and with the modified force field were very similar (Table 1). Overall, our results indicate that phase separation and the mechanism of its stabilization by aromatics are robust with respect to reasonable variations in cholesterol-aromatic interaction.

## Discussion

Membrane lateral organization has paramount importance in cellular processes such as signaling, protein trafficking, and viral infection. Perturbations of membrane lateral organization can affect a large number of processes vital to the cell. Changes in domain structure can be brought about by modifications in membrane composition or by the addition of molecules that dissolve in the membrane. The effect of a few small molecules (alcohols [9], [10], surfactants [17], anesthetics [9], [10]) on membrane lateral organization has been studied experimentally in model systems. It has been observed that some molecules stabilize domains, while others destabilize them, but results are sparse, so it has not been possible to pinpoint the chemical or physical properties determining stabilization and destabilization of domains. Moreover, little is known on the mechanisms of lipid domain reshaping. Both the thermodynamics and the mechanisms of domain reshaping are difficult to study in living cells because of the small size of the domains and their highly dynamic nature.

Here we studied the effect of a set of common hydrophobic molecules on the lateral organization of model lipid membranes, consisting of saturated and unsaturated phospholipids, and cholesterol. Such membranes display clear phase separation between L_d_ and L_o_ phases at room temperature, and lipid mixing at higher temperatures – both experimentally and in MARTINI CG simulations. Our simulations predict that common hydrophobic compounds have major effects on lipid mixing in model membranes. Based on their effect on phase separation, the hydrophobic molecules selected for our study can be divided in two groups: (1) octane, hexadecane, and cyclohexane distribute preferentially at domain boundaries and destabilize phase separation; (2) benzene, fullerene, and polystyrene, instead, partition largely to the L_d_ phase and stabilize phase separation. These predictions can be tested directly with experiments on model systems. Considering the diversity of the chemical structures used in our study, our conclusions are likely to be valid in a general way for purely hydrophobic compounds.

The persistence of phase separation in the presence of aromatic compounds at high temperature raises questions on the possibility that the systems might be trapped in metastable states. Based on the analysis of contact fractions, convergence requires about 1 µs in all simulated systems. Since our sampling is generally at least one order of magnitude longer, we expect phase separation to be well converged in all simulations. Yet, to guarantee that our simulations overcome potential metastable states, we repeated one simulation with benzene (high concentration, high temperature) starting from a well-mixed membrane (see Methods for details). Again, we observed phase separation within about 1 µs, and the contact fractions converged rapidly to the same values calculated in the original set of simulations (see Table 1). We conclude that persistence of phase separation in simulations with aromatic compounds is not due to limited sampling or the presence of metastable states.

Coarse-grained simulations provide both equilibrium and time-dependent distributions of all species in a membrane; therefore they can be used to shed light on the mechanism of action of the different compounds – which is more difficult to access experimentally. For the first group of molecules (octane, hexadecane, and cyclohexane), we found that the mechanism of action is the one typical for linactants: those compounds tend to accumulate in the L_d_-L_o_ interface region, which leads to a destabilization of the phase boundary [26]. For the second group (benzene, fullerene, and polystyrene), crowding of the L_d_ phase prevents cholesterol from entering it, causing enrichment in cholesterol in the L_o_ phase, particularly at high temperature. Cholesterol distribution in the L_o_ phase has been associated to phase stabilization [27].

One of the goals of our study was to understand which chemical and physical properties of hydrophobic molecules determine their effect on domain stability. The compounds used in our study differ in several ways. Octane and hexadecane differ only in size, and they differ from the other compounds for the absence of ring structures. Hexadecane and cyclohexane are smaller than fullerene and polystyrene but bigger than benzene. Clearly the difference in domain remodeling behavior does not depend on the size of the solute. Nor it depends on cyclic nature of the compounds: both benzene and cyclohexane are cyclic (and of very similar size), but they have opposite effects on domain stabilization. Instead, the main discriminant between the two groups of compounds is aromaticity. The stronger affinity of aromatic compounds for the L_d_ phase can be explained by π-π interactions between aromatic rings and double bonds in unsaturated acyl chains – which are captured by the force field in an effective way, through more attractive Lennard-Jones interactions. Aliphatic compounds, on the contrary, have higher affinity for saturated acyl chains (as expected based on experimental partitioning data [28]) but the dense packing of the L_o_ phase prevents mixing of these solutes with the L_o_ phase, as shown before for transmembrane peptides [29]. Dense packing of the L_o_ phase appears to be responsible for preferential partitioning of aliphatic compounds at domain boundaries.

Together with the current study, there is a growing body of evidence indicating that small molecules can have a pronounced effect on lipid phase behavior. Like octane, hexadecane, and cyclohexane, also amphipathic molecules such as palmitoyloleoyl-phosphatidylcholine (POPC) [16] and vitamin E [17] distribute at the L_o_-L_d_ interface and destabilize domains. Stabilization of phase separation has also been reported, for instance, in our previous work on polystyrene fragments of varying sizes [19], but also for transmembrane peptides [18] and for less hydrophobic solutes such as benzyl alcohol [17]. Exclusion of cholesterol from the L_d_ phase due to crowding is likely to be the underlying mechanism of domain stabilization in all of these cases.

### Conclusions

In conclusion, we showed that relatively small concentrations of six different hydrophobic compounds have a major impact on lipid domain stability in model membranes. Aliphatic compounds behave like linactants, accumulating at the interface between liquid-ordered and liquid-disordered domains and promoting lipid mixing, while aromatic compounds partition preferentially to liquid-disordered domains and stabilize phase separation. Both stabilization and destabilization of lipid domains can have an important impact on biological function. For example, it has been shown that, *in vitro*, raft-disrupting drugs can inhibit various cellular signaling pathways, including apoptitic pathways [30]. More studies are needed to understand how the complex interplay between lipids, proteins, and drugs affects signaling pathways *in vivo*. Nevertheless, our results on model systems shed light on the driving forces and the mechanisms of domain perturbation, and can be used to guide the rational design of drugs modulating phase separation. Knowledge of how hydrophobic molecules affect phase separation can also help understanding the side effects of drugs, and suggest possible mechanisms behind the toxicity of hydrophobic pollutants, such as hydrocarbons, air-borne carbon nanoparticles and nanoplastics.

## Methods

### System setup

We carried out all MD simulations at the coarse-grained (CG) level using the MARTINI force field [20], [21], [31]. The MARTINI force field is widely used for a large variety of membrane processes, including domain formation, as reviewed in refs [32] and [33]. We carried out simulations of lipid mixtures in water, containing 540 DLiPC, 828 DPPC, and 576 cholesterol molecules, as well as 21,880 water particles. The membrane was originally formed through self-assembly, by Risselada *et al.* [22] In the absence of solutes, at 295 K, the membranes yield phase separation and display a liquid-ordered (L_o_) and a liquid-disordered (L_d_) phase, which form stripes across the periodic box (Fig. 1).

We then simulated the same model membrane in the presence of six different hydrophobic solutes: octane, hexadecane, cyclohexane, benzene, C_60_ fullerene, and polystyrene. We carried out the simulations at two solute concentrations. For the lower concentration, we used a constant solute∶membrane molar ratio of 3.3% (*i.e.* 64 solute molecules). For the higher concentration, we used a constant mass ratio of 4.8% (based on real molecular masses). Except for the simulations with polystyrene, simulations started with the solute evenly distributed, on a grid, at the center of the membrane. The simulation time was between 6 and 30 µs (see Table 1). For polystyrene, we used simulations from a previous work by Rossi *et al.* [19]; in this case, the system contained 6 chains of 100 styrene residues each (PS100). PS100 chains formed compact clusters in water but dissolved once in the membrane interior, on a time scale of about 10 µs. In addition to the simulations above, some systems were simulated also at higher temperature or with additional solute concentrations. Simulations at higher temperatures were usually started from the same starting configurations used in simulations at lower temperature. One additional simulation was carried out in the presence of benzene starting with lipids completely mixed; in this case, the starting configuration was taken from the simulation with cyclohexane at 325 K, which showed a very high degree of mixing. Considering all simulations, the total sampling was over 680 µs. A list of all simulations performed is reported in Table 1.

### Simulation parameters

The MARTINI [20], [21] force field was used in all simulations. For simulations with fullerene, we used the fullerene model developed by Monticelli [24], [34]. For simulations with polystyrene, we used the model by Rossi *et al.* [35]. One simulation was carried out with a modified force field, in which the strength of cholesterol-aromatic interaction was increased; namely, the SC4-SC1 interaction was increased from 3.1 kJ/mol to ε = 3.5 kJ/mol, while leaving all other interactions unchanged. Non-bonded interactions were calculated with a cut-off of 1.2 nm, on which we applied a shift function, starting at 0.9 nm for Van der Waals interactions and at 0 nm for Coulomb interactions. Charges were screened with a relative dielectric constant ε_rel_ = 15. A neighbor list (with a cut-off of 1.3 nm) was updated every 10 steps.

Simulations were run in the NPT ensemble. Pressure was coupled to 1 bar using a semi-isotropic barostat and the Parrinello-Rahman algorithm [36] (time constant of 4 ps and compressibility of 4.5×10^−5^ bar^−1^). The temperature was coupled using the Bussi-Donadio-Parrinello thermostat [37] (time constant of 2 ps). We carried out most simulations at 295 K, and some additional ones at higher temperatures: 305 K, 315 K, and 325 K (see Table 1). We used the leapfrog integrator and an integration time step of 20 fs. The time step was reduced to 15 fs in simulations at temperatures of 315 K or higher, and 18 fs in all simulations with polystyrene. All simulations were carried out using the GROMACS software package (v4.5) [38].

### Simulation analysis

#### Contact fraction

As a metric for phase separation, we used DLiPC-DPPC contact fractions, defined as:
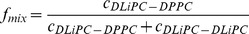
where *c* is the number of contacts between the two lipid species in subscript. Contacts were calculated only between PO4 beads of the lipids. We used a distance threshold of 1.1 nm, like in previous work by Domanski [18].

Solute and cholesterol lateral distribution were quantified by calculating solute-DLiPC and cholesterol-DLiPC contact fraction, respectively. These contact fractions are defined as:
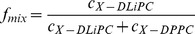
where X is either solute or cholesterol, and *c* is the number of contacts between the species in subscript. Contacts between cholesterol and DLiPC were calculated using only the PO4 bead of lipids and the ROH bead of cholesterol, with a distance threshold of 1.1 nm. For the solute-DLiPC contact fraction, we used all particles of the lipids and the solute, and a distance threshold of 0.8 nm. Averaging was done over the last 5 µs of each simulation, and errors were estimated by block averaging as implemented in GROMACS [38].

#### Density landscapes

We used two kinds of density landscapes to visualize the density of the different molecules in the plane of the membrane: the partial density landscape, and the DLiPC density fraction landscape. The partial density landscape was defined as the density of a given molecule calculated on a grid placed in the plane of the membrane (XY plane). The X and Y dimensions were divided in 50 bins each so the grid cells were about 0.4×0.4 nm. We averaged densities over the last 0.5 µs of the simulations. The DLiPC density fraction was defined as the fraction of DLiPC density over the total density of PC lipids for each cell; therefore, it can assume values between 0 (DPPC is the only lipid in that cell) and 1 (DLiPC is the only lipid type in that cell). The main interfaces are located where the DLiPC density fraction is 0.5. Landscapes were calculated using an in-house software freely available from our website (http://perso.ibcp.fr/luca.monticelli, see also ref [39]).

#### Thickness calculations

Membrane thickness was calculated as the distance between the average positions of PO4 beads of the two leaflets on a grid. The X and Y dimensions were divided in 50 bins each, so the grid cells were about 0.4×0.4 nm^2^. For each cell, the thickness was averaged over the last 0.5 µs for each trajectory. The thickness of a phase in simulations with stripe domains was defined as the most frequent local thickness in the thickness landscape. Thicknesses were calculated with an in-house software freely available on our website (http://perso.ibcp.fr/luca.monticelli/).

## Supporting Information

Figure S1 **Mechanism of action of linactants.** Lipid mixing (as DLiPC-DPPC contact fraction) as a function of solute phase distribution (as solute-DLiPC contact fraction) along time (color scale, from green to blue). The vertical plain line marks a solute-DLiPC contact fraction of 0.5, that is the value expected when the solute is at the Lo-Ld interface. The vertical dashed line marks a solute-DLiPC contact fraction of 0.39, that is the estimated value for ideal mixing. The horizontal solid line is the DLiPC-DPPC contact fraction in the reference simulation, in the absence of solute.
(TIFF)

Figure S2 **Mechanism of action of aromatic compounds.** (a) Solute distribution (expressed as solute-DLiPC contact fraction) for aromatic compounds at different concentrations and temperatures. “L” stands for low concentration and “H” stands for high concentration. All aromatic compounds show a strong preference for unsaturated lipids. (b) Cholesterol distribution (expressed as cholesterol-DLiPC contact fraction) at 295 K. The vertical solid line indicates the value observed in the absence of solute; the dashed lines represent error estimates.
(TIFF)

Figure S3 **Difference in thickness between L_o_ and L_d_ phases.** Left panels: histograms of membrane thickness in the absence and in the presence of different solutes. Thicknesses are calculated separately for the DLiPC and the DPPC components, based on the distance (along the bilayer normal) between phosphate groups in each leaflet. Line colors are the same as in the right panels. Right panels: difference in thickness between the DLiPC-rich and the DPPC-rich phases, in the absence and in the presence of different solutes. Numbers in parentheses indicate different replicas of the simulations.
(TIFF)

Figure S4 **Robustness of the results.** Snapshots of a single leaflet from simulations of systems with high concentration of benzene at 325 K, carried out with the original MARTINI force field (left panel) and the modified force field (right panel). Colors are the same as in Fig. 1: DPPC is colored in blue and DLiPC in red.
(TIFF)

## References

[1] Simons K, Ikonen E (1997) Functional rafts in cell membranes. Nature 387: 569–572. 9177342 10.1038/42408

[2] Chamberlain LH, Burgoyne RD, Gould GW (2001) SNARE proteins are highly enriched in lipid rafts in PC12 cells: Implications for the spatial control of exocytosis. Proceedings of the National Academy of Sciences of the United States of America 98: 5619–5624. 11331757 10.1073/pnas.091502398 PMC33262

[3] Lang T, Bruns D, Wenzel D, Riedel D, Holroyd P, et al (2001) SNAREs are concentrated in cholesterol-dependent clusters that define docking and fusion sites for exocytosis. EMBO J 20: 2202–2213. 11331586 10.1093/emboj/20.9.2202 PMC125434

[4] Simons K, Ikonen E (2000) Cell biology - How cells handle cholesterol. Science 290: 1721–1726. 11099405 10.1126/science.290.5497.1721

[5] Lundbaek JA, Andersen OS, Werge T, Nielsen C (2003) Cholesterol-induced protein sorting: An analysis of energetic feasibility. Biophysical Journal 84: 2080–2089. 12609909 10.1016/S0006-3495(03)75015-2 PMC1302776

[6] Suomalainen M (2002) Lipid Rafts and Assembly of Enveloped Viruses. Traffic 3: 705–709. 12230468 10.1034/j.1600-0854.2002.31002.x

[7] Suzuki T, Suzuki Y (2006) Virus Infection and Lipid Rafts. Biological and Pharmaceutical Bulletin 29: 1538–1541. 16880600 10.1248/bpb.29.1538

[8] Simons K, Ehehalt R (2002) Cholesterol, lipid rafts, and disease. Journal of Clinical Investigation 110: 597–603. 12208858 10.1172/JCI16390 PMC151114

[9] Weinrich M, Worcester DL (2013) Xenon and Other Volatile Anesthetics Change Domain Structure in Model Lipid Raft Membranes. Journal of Physical Chemistry B 10.1021/jp411261g PMC3914297 24299622

[10] Gray E, Karslake J, Machta BB, Veatch SL (2013) Liquid General Anesthetics Lower Critical Temperatures in Plasma Membrane Vesicles. Biophysical Journal 105: 2751–2759. 24359747 10.1016/j.bpj.2013.11.005 PMC3882514

[11] Simons K, Vaz WLC (2004) Model Systems, Lipid Rafts, and Cell Membranes. Annual Review of Biophysics and Biomolecular Structure 33: 269–295. 10.1146/annurev.biophys.32.110601.141803 15139814

[12] Veatch SL, Keller SL (2003) Separation of liquid phases in giant vesicles of ternary mixtures of phospholipids and cholesterol. Biophysical Journal 85: 3074–3083. 14581208 10.1016/S0006-3495(03)74726-2 PMC1303584

[13] Ingolfsson HI, Andersen OS (2010) Screening for Small Molecules' Bilayer-Modifying Potential Using a Gramicidin-Based Fluorescence Assay. Assay and Drug Development Technologies 8: 427–436. 20233091 10.1089/adt.2009.0250 PMC2929145

[14] Lucio M, Lima JLFC, Reis S (2010) Drug-Membrane Interactions: Significance for Medicinal Chemistry. Current Medicinal Chemistry 17: 1795–1809. 20345343 10.2174/092986710791111233

[15] Sikkema J, Debont JAM, Poolman B (1995) Mechanisms of membrane toxicity of hydrocarbons. Microbiological Reviews 59: 201–222. 7603409 10.1128/mr.59.2.201-222.1995 PMC239360

[16] Schafer LV, Marrink SJ (2010) Partitioning of Lipids at Domain Boundaries in Model Membranes. Biophysical Journal 99: L91–L93. 21156123 10.1016/j.bpj.2010.08.072 PMC3000497

[17] Muddana HS, Chiang HH, Butler PJ (2012) Tuning Membrane Phase Separation Using Nonlipid Amphiphiles. Biophysical Journal 102: 489–497. 22325271 10.1016/j.bpj.2011.12.033 PMC3274828

[18] Domanski J, Marrink SJ, Schafer LV (2012) Transmembrane helices can induce domain formation in crowded model membranes. Biochimica et Biophysica Acta-Biomembranes 1818: 984–994. 10.1016/j.bbamem.2011.08.021 21884678

[19] Rossi G, Barnoud J, Monticelli L (2014) Polystyrene Nanoparticles Perturb Lipid Membranes. Journal of Physical Chemistry Letters 5: 241–246. 26276207 10.1021/jz402234c

[20] Marrink SJ, de Vries AH, Mark AE (2004) Coarse grained model for semiquantitative lipid simulations. Journal of Physical Chemistry B 108: 750–760.

[21] Marrink SJ, Risselada HJ, Yefimov S, Tieleman DP, de Vries AH (2007) The MARTINI force field: Coarse grained model for biomolecular simulations. Journal of Physical Chemistry B 111: 7812–7824. 10.1021/jp071097f 17569554

[22] Risselada HJ, Marrink SJ (2008) The molecular face of lipid rafts in model membranes. Proceedings of the National Academy of Sciences of the United States of America 105: 17367–17372. 18987307 10.1073/pnas.0807527105 PMC2579886

[23] MacCallum JL, Tieleman DP (2006) Computer simulation of the distribution of hexane in a lipid bilayer: spatially resolved free energy, entropy, and enthalpy profiles. Journal of the American Chemical Society 128: 125–130. 16390139 10.1021/ja0535099

[24] Wong-Ekkabut J, Baoukina S, Triampo W, Tang IM, Tieleman DP, et al (2008) Computer simulation study of fullerene translocation through lipid membranes. Nature Nanotechnology 3: 363–368. 10.1038/nnano.2008.130 18654548

[25] Barnoud J, Rossi G, Monticelli L (2014) Lipid membranes as solvents for carbon nanoparticles. Physical Review Letters 112: 068102. 24580709 10.1103/PhysRevLett.112.068102

[26] Trabelsi S, Zhang S, Lee TR, Schwartz DK (2008) Linactants: Surfactant analogues in two dimensions. Physical Review Letters 100: 037802. 18233038 10.1103/PhysRevLett.100.037802

[27] Silvius JR (2003) Role of cholesterol in lipid raft formation: lessons from lipid model systems. Biochimica et Biophysica Acta-Biomembranes 1610: 174–183. 10.1016/s0005-2736(03)00016-6 12648772

[28] Abraham MH, Whiting GS, Fuchs R, Chambers EJ (1990) Thermodynamics of solute transfer from water to hexadecane. Journal of the Chemical Society-Perkin Transactions 2: 291–300.

[29] Schafer LV, de Jong DH, Holt A, Rzepiela AJ, de Vries AH, et al (2011) Lipid packing drives the segregation of transmembrane helices into disordered lipid domains in model membranes. Proceedings of the National Academy of Sciences of the United States of America 108: 1343–1348. 21205902 10.1073/pnas.1009362108 PMC3029762

[30] George KS, Wu S (2012) Lipid raft: A floating island of death or survival. Toxicology and Applied Pharmacology 259: 311–319. 22289360 10.1016/j.taap.2012.01.007 PMC3299927

[31] Monticelli L, Kandasamy SK, Periole X, Larson RG, Tieleman DP, et al (2008) The MARTINI coarse-grained force field: Extension to proteins. Journal of Chemical Theory and Computation 4: 819–834. 26621095 10.1021/ct700324x

[32] Marrink SJ, Tieleman DP (2013) Perspective on the Martini model. Chemical Society Reviews 42: 6801–6822. 23708257 10.1039/c3cs60093a

[33] Baoukina S, Mendez-Villuendas E, Bennett WFD, Tieleman DP (2013) Computer simulations of the phase separation in model membranes. Faraday Discussions 161: 63–75. 23805738 10.1039/c2fd20117h

[34] Monticelli L (2012) On Atomistic and Coarse-Grained Models for C60 Fullerene. Journal of Chemical Theory and Computation 8: 1370–1378. 26596752 10.1021/ct3000102

[35] Rossi G, Monticelli L, Puisto SR, Vattulainen I, Ala-Nissila T (2011) Coarse-graining polymers with the MARTINI force-field: polystyrene as a benchmark case. Soft Matter 7: 698–708.

[36] Parrinello M, Rahman A (1981) Polymorphic Transitions in Single Crystals - a New Molecular Dynamics Method. Journal of Applied Physics 52: 7182–7190.

[37] Bussi G, Donadio D, Parrinello M (2007) Canonical sampling through velocity rescaling. Journal of Chemical Physics 126: 014101. 17212484 10.1063/1.2408420

[38] Hess B, Kutzner C, van der Spoel D, Lindahl E (2008) GROMACS 4: Algorithms for highly efficient, load-balanced, and scalable molecular simulation. Journal of Chemical Theory and Computation 4: 435–447. 26620784 10.1021/ct700301q

[39] Castillo N, Monticelli L, Barnoud J, Tieleman DP (2013) Free energy of WALP23 dimer association in DMPC, DPPC, and DOPC bilayers. Chemistry and Physics of Lipids 169: 95–105. 23415670 10.1016/j.chemphyslip.2013.02.001

