# Supplementary figures and images for: Hydrophobic Compounds Reshape Membrane Domains
Source: PLoS Comput Biol. 2014 Oct 9;10(10):e1003873. doi: 10.1371/journal.pcbi.1003873 (PMC4191877; doi:10.1371/journal.pcbi.1003873)

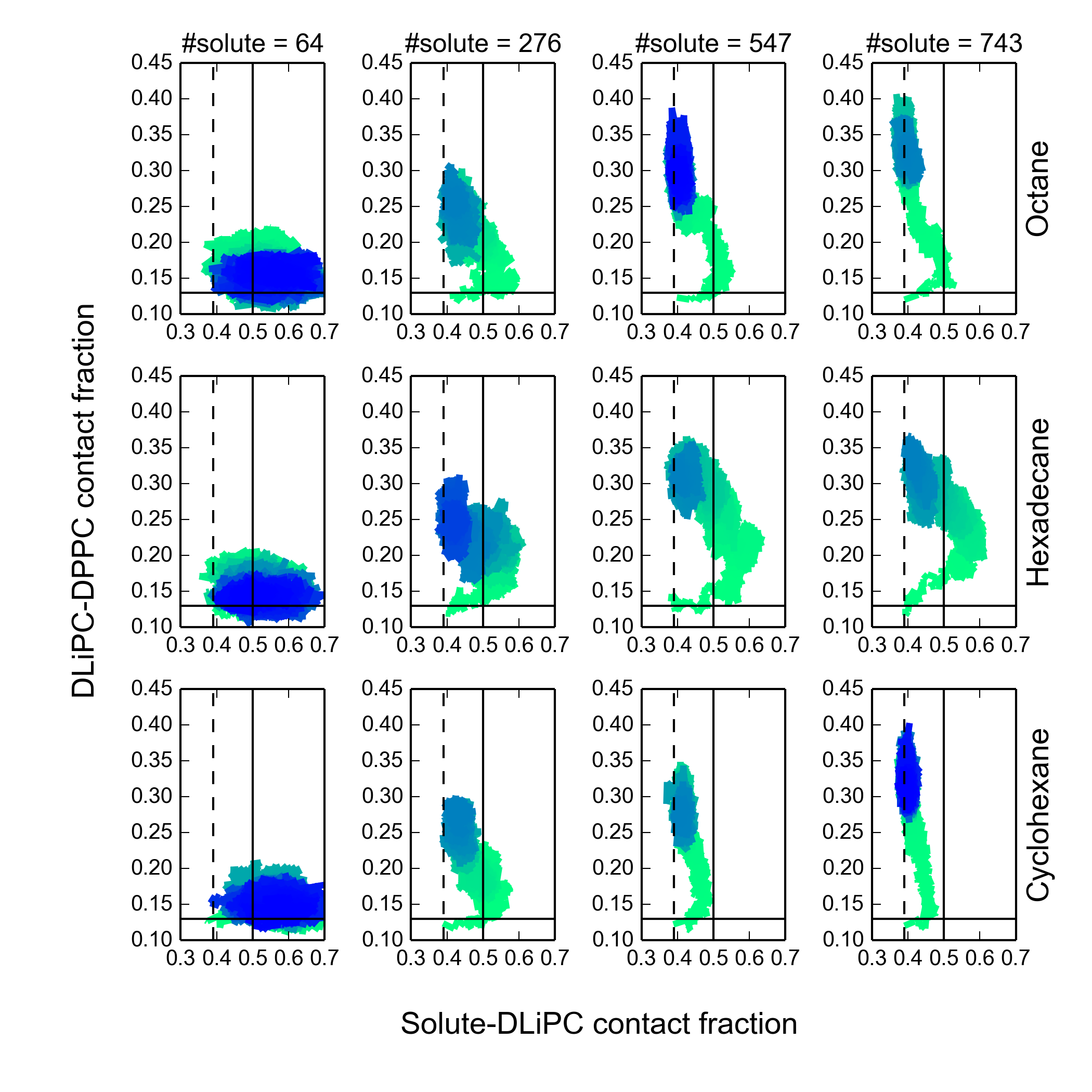

Supplement: Figure S1 — Mechanism of action of linactants. Lipid mixing (as DLiPC-DPPC contact fraction) as a function of solute phase distribution (as solute-DLiPC contact fraction) along time (color scale, from green to blue). The vertical plain line marks a solute-DLiPC contact fraction of 0.5, that is the value expected when the solute is at the Lo-Ld interface. The vertical dashed line marks a solute-DLiPC contact fraction of 0.39, that is the estimated value for ideal mixing. The horizontal solid line is the DLiPC-DPPC contact fraction in the reference simulation, in the absence of solute. (TIFF) [file pcbi.1003873.s001.tiff]

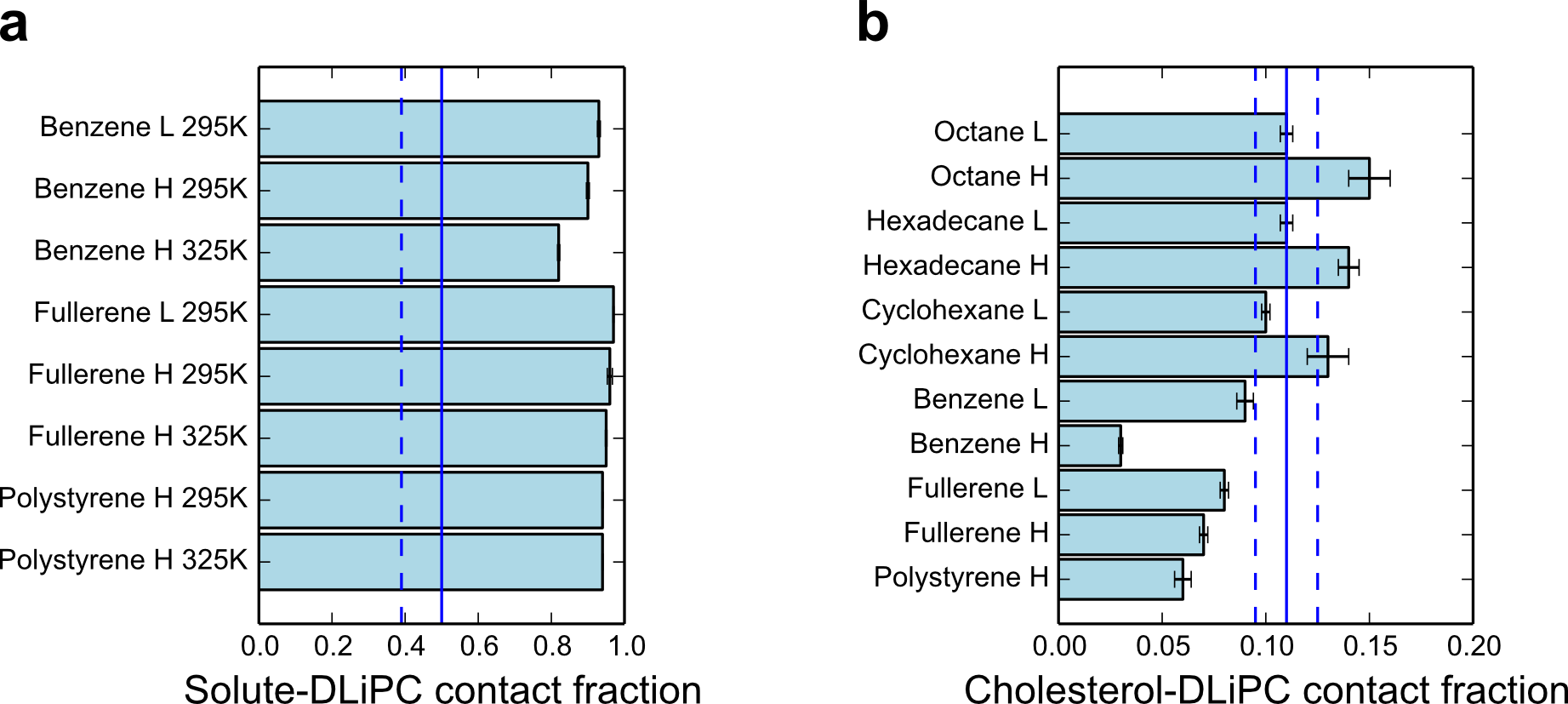

Supplement: Figure S2 — Mechanism of action of aromatic compounds. (a) Solute distribution (expressed as solute-DLiPC contact fraction) for aromatic compounds at different concentrations and temperatures. “L” stands for low concentration and “H” stands for high concentration. All aromatic compounds show a strong preference for unsaturated lipids. (b) Cholesterol distribution (expressed as cholesterol-DLiPC contact fraction) at 295 K. The vertical solid line indicates the value observed in the absence of solute; the dashed lines represent error estimates. (TIFF) [file pcbi.1003873.s002.tiff]

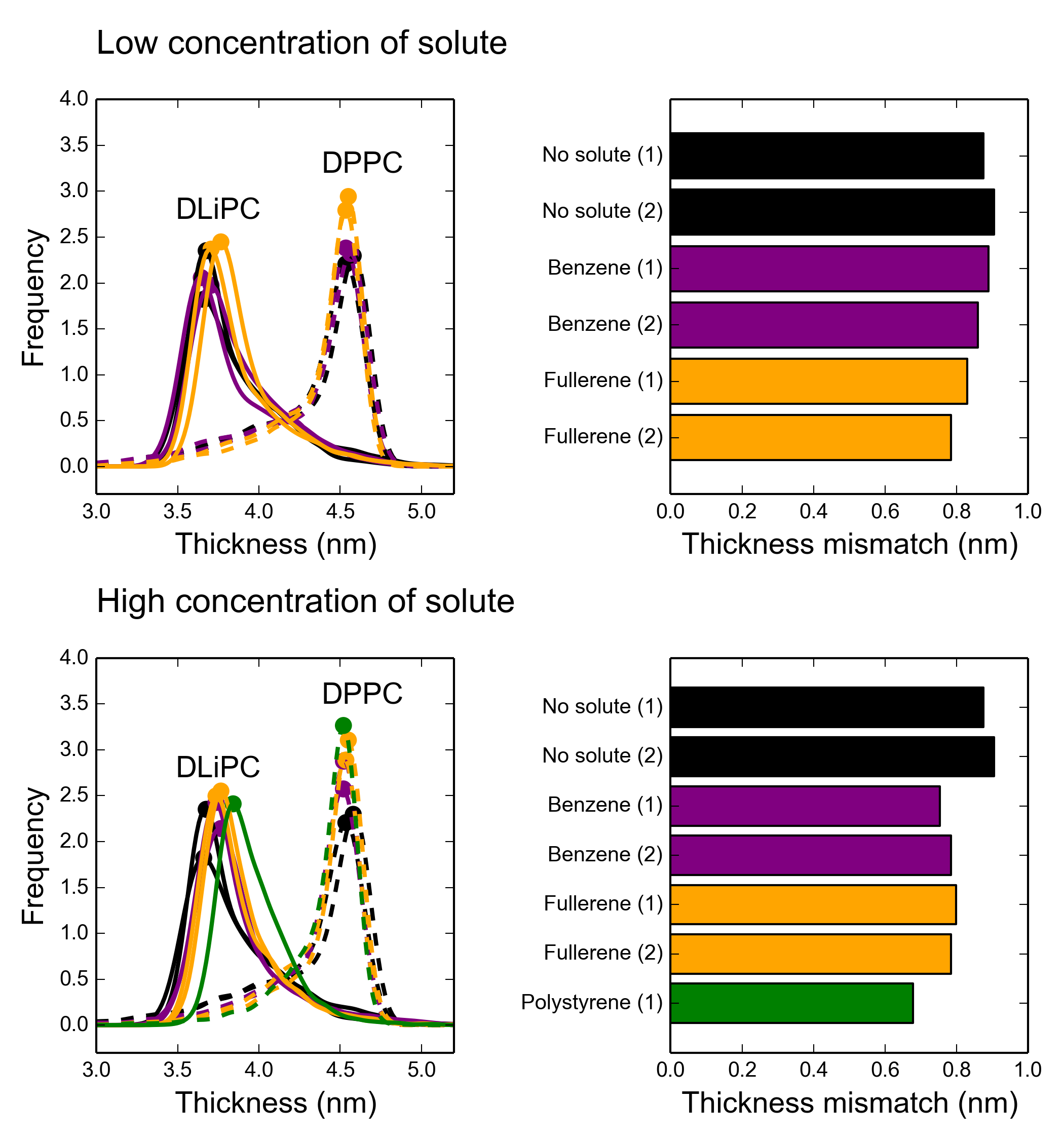

Supplement: Figure S3 — Difference in thickness between Lo and Ld phases. Left panels: histograms of membrane thickness in the absence and in the presence of different solutes. Thicknesses are calculated separately for the DLiPC and the DPPC components, based on the distance (along the bilayer normal) between phosphate groups in each leaflet. Line colors are the same as in the right panels. Right panels: difference in thickness between the DLiPC-rich and the DPPC-rich phases, in the absence and in the presence of different solutes. Numbers in parentheses indicate different replicas of the simulations. (TIFF) [file pcbi.1003873.s003.tiff]

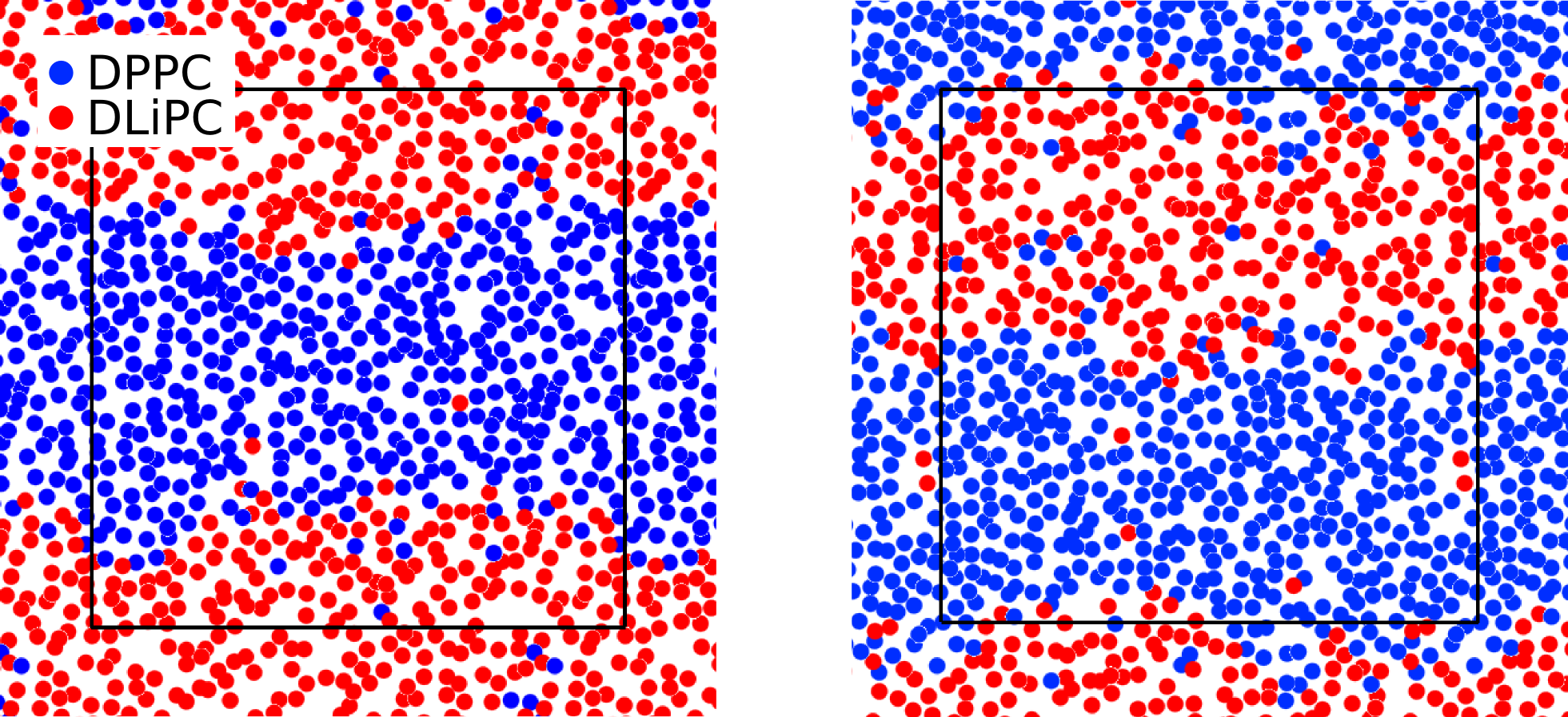

Supplement: Figure S4 — Robustness of the results. Snapshots of a single leaflet from simulations of systems with high concentration of benzene at 325 K, carried out with the original MARTINI force field (left panel) and the modified force field (right panel). Colors are the same as in Fig. 1: DPPC is colored in blue and DLiPC in red. (TIFF) [file pcbi.1003873.s004.tiff]
